# Cell-free DNA TAPS provides multimodal information for early cancer detection

**DOI:** 10.1126/sciadv.abh0534

**Published:** 2021-09-01

**Authors:** Paulina Siejka-Zielińska, Jingfei Cheng, Felix Jackson, Yibin Liu, Zahir Soonawalla, Srikanth Reddy, Michael Silva, Luminita Puta, Misti Vanette McCain, Emma L. Culver, Noor Bekkali, Benjamin Schuster-Böckler, Pier Francesco Palamara, Derek Mann, Helen Reeves, Eleanor Barnes, Shivan Sivakumar, Chun-Xiao Song

**Affiliations:** 1 Ludwig Institute for Cancer Research, Nuffield Department of Medicine, University of Oxford, Oxford, UK.; 2 Target Discovery Institute, Nuffield Department of Medicine, University of Oxford, Oxford, UK.; 3 Department of Computer Science, University of Oxford, Oxford, UK.; 4 Oxford University Hospitals NHS Foundation Trust, Oxford, UK.; 5 Oxford Transplant Centre, Churchill Hospital, Oxford, UK.; 6 Department of HPB Surgery, Oxford University Hospitals NHS Foundation Trust, Oxford, UK.; 7 Newcastle University Translational and Clinical Research Institute, The Medical School, Newcastle University, Newcastle upon Tyne, UK.; 8 Newcastle University Centre for Cancer, The Medical School, Newcastle University, Newcastle upon Tyne, UK.; 9 Peter Medawar Building and Translational Gastroenterology Unit, Nuffield Department of Medicine, University of Oxford, Oxford, UK.; 10 Department of Gastroenterology, John Radcliffe Hospital, Oxford University Hospitals NHS Trust, Oxford, UK.; 11 Big Data Institute, University of Oxford, Oxford, UK.; 12 Department of Statistics, University of Oxford, Oxford, UK.; 13 Wellcome Centre for Human Genetics, University of Oxford, Oxford, UK.; 14 Newcastle Fibrosis Research Group, Biosciences Institute, Faculty of Medical Sciences, Newcastle University, Newcastle upon Tyne, UK.; 15 Fibrofind, Medical School, Newcastle University, Newcastle upon Tyne, UK.; 16 Liver Unit, Freeman Hospital, Newcastle upon Tyne Hospitals NHS Foundation Trust, Newcastle upon Tyne, UK.; 17 Department of Oncology, University of Oxford, Oxford, UK.; 18 Department of Oncology, Oxford University Hospitals NHS Foundation Trust, Oxford, UK.; 19 Kennedy Institute of Rheumatology, University of Oxford, Oxford, UK.

## Abstract

Multimodal, genome-wide characterization of epigenetic and genetic information in circulating cell-free DNA (cfDNA) could enable more sensitive early cancer detection, but it is technologically challenging. Recently, we developed TET-assisted pyridine borane sequencing (TAPS), which is a mild, bisulfite-free method for base-resolution direct DNA methylation sequencing. Here, we optimized TAPS for cfDNA (cfTAPS) to provide high-quality and high-depth whole-genome cell-free methylomes. We applied cfTAPS to 85 cfDNA samples from patients with hepatocellular carcinoma (HCC) or pancreatic ductal adenocarcinoma (PDAC) and noncancer controls. From only 10 ng of cfDNA (1 to 3 ml of plasma), we generated the most comprehensive cfDNA methylome to date. We demonstrated that cfTAPS provides multimodal information about cfDNA characteristics, including DNA methylation, tissue of origin, and DNA fragmentation. Integrated analysis of these epigenetic and genetic features enables accurate identification of early HCC and PDAC.

## Teaser

Novel cell-free DNA whole-genome methylome sequencing method enables accurate cancer detection.

## INTRODUCTION

Although recent advances in cancer research offer new ways to treat cancer, early detection still represents the best opportunity for curing cancer. Early-stage treatment not only greatly improves patient survival but also costs considerably less. Circulating cell-free DNA (cfDNA)—the free-floating DNA in blood plasma originating from cell death in various healthy and diseased tissues—holds tremendous potential to develop an early cancer detection assay (*1*). Genetic information in cfDNA, such as mutations and copy number variations (CNVs), demonstrate potential utility for monitoring cancer progression and treatment (*2*–*4*). However, genetic alterations are challenging to detect given the low fraction of tumor DNA in early-stage disease (*5*). Furthermore, genetic alterations are weakly informative about the tissue of origin needed to determine the location of malignancy (*6*).

In contrast, widespread epigenetic changes such as DNA methylation of both cancer cells and tumor microenvironment occur early in tumorigenesis (*7*). Recent studies have shown cfDNA methylation to be one of the most promising biomarkers for early cancer detection by providing thousands of methylation changes that can be combined to overcome detection limits and tissue-of-origin information that allows cancer localization with high confidence (*8*–*16*). DNA methylation is best determined by a whole-genome, base-resolution, and quantitative sequencing method, such as bisulfite sequencing. However, bisulfite sequencing is DNA damaging and expensive, so current cfDNA methylation sequencing is limited by being low-depth (*10*, *11*, *14*), targeted (*8*, *12*, *13*, *15*), or low-resolution and qualitative enrichment–based sequencing (*9*), thus imperfectly capturing the cfDNA methylome.

Recently, we developed TET-assisted pyridine borane sequencing (TAPS), a new bisulfite-free DNA methylation sequencing method (*17*). TAPS used mild chemistry to detect DNA methylation directly and showed improved sequence quality, mapping rate, and coverage compared to bisulfite sequencing, while reducing sequencing cost by half (*17*). The combination of direct methylation detection and the nondestructive nature of TAPS makes it ideal not only for DNA methylation analysis but also for simultaneous genetic analysis in cfDNA, which could enhance noninvasive cancer detection by liquid biopsies (*18*). Here, we optimized TAPS for cfDNA (cfTAPS) to deliver high-quality and high-depth whole-genome methylome from only 10 ng of cfDNA.

We applied cfTAPS to hepatocellular carcinoma (HCC) and pancreatic ductal adenocarcinoma (PDAC) cfDNA, two cancer types with particularly poor prognosis mostly due to detection at an advanced disease stage (*19*, *20*). Noninvasive methods for early detection of PDAC and HCC are not available, which contributes to their late diagnosis. For decades, HCC detection has relied on liver ultrasound, combined with serum α-fetoprotein (AFP) measurements (*21*). However, these methods have low specificity and sensitivity (*21*, *22*). There is no blood test to detect or diagnose PDAC. Carbohydrate antigen 19-9 (CA19-9) is used for monitoring PDAC treatment and development, but its sensitivity and specificity are too low to diagnose or screen for PDAC (*23*). Therefore, novel approaches for PDAC and HCC detection are urgently needed.

Here, we demonstrated that the rich information from cfTAPS enables integrated multimodal epigenetic and genetic analysis of differential methylation, tissue of origin, and fragmentation profiles to accurately distinguish cfDNA samples from patients with HCC and PDAC from controls and patients with precancerous inflammatory conditions.

## RESULTS

### Adaptation of cfTAPS sequencing

**Fig. 1.**
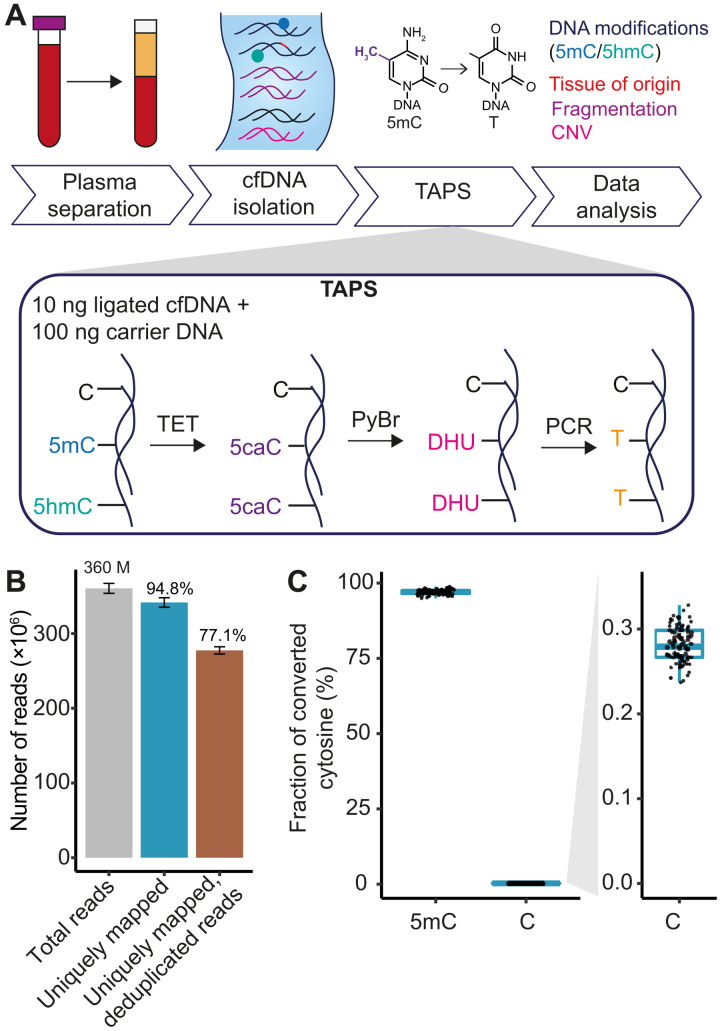
cfDNA analysis by TAPS. (**A**) Schematic representation of the TAPS approach for cfDNA analysis. CfDNA is isolated from 1 to 3 ml of plasma. cfDNA (10 ng) is ligated to Illumina sequencing adapters and topped up with 100 ng of carrier DNA. Subsequently, 5mC and 5hmC in DNA are oxidized by mTet1CD enzyme to 5caC, reduced by PyBr to DHU, and amplified and detected as T in the final sequencing. Computational analysis of TAPS data allows simultaneous characterization of multiple cfDNA features including DNA methylation, tissue of origin, fragmentation patterns, and CNVs. (**B**) Number of total reads, uniquely mapped reads, and uniquely mapped, PCR deduplicated reads in 87 cfDNA TAPS libraries. Total number of reads, mean percentage of uniquely mapped reads, and deduplicated reads compared to total reads are shown above the bars. Error bars represent SE. (**C**) 5mC conversion rate and false-positive rate in 85 cfDNA TAPS libraries based on spike-in controls with modified or unmodified cytosines at the known positions. Each dot represents an individual sample.

We first optimized the TAPS protocol to work with low-input cfDNA (10 ng, purified from 1 to 3 ml of plasma). Briefly, 10 ng of cfDNA is first ligated to Illumina adapters and 100 ng of carrier DNA is then added to the sample before TET oxidation and pyridine borane (PyBr) reduction steps (Fig. 1A). We found that the addition of carrier DNA improves the recovery of cfDNA during the workflow and results in higher library yields when compared to the standard TAPS protocol (fig. S1A) (*17*). Subsequently, 5-methylcytosine (5mC) and 5-hydroxymethylcytosine (5hmC) in cfDNA are oxidized by mTet1CD enzyme to 5-carboxylcytosine (5caC) and reduced to dihydrouracil (DHU), which is amplified as T in the final polymerase chain reaction (PCR) step (Fig. 1A).

We applied cfTAPS to 87 cfDNA samples. Libraries were sequenced to a mean of 360 million read pairs (11.6× mean depth, range 8.2 to 22×) and resulted in high unique mapping rate and unique deduplicated mapping rate of 94.8 and 77.1%, respectively (Fig. 1B and table S1). Among the mapped reads, 99.95% were mapped to the human genome (fig. S1B). In comparison, a recent cfDNA whole-genome bisulfite sequencing (WGBS) study (*24*) sequenced to a similar depth (a mean of 371 million read pairs) and resulted in significantly lower unique mapping rate (63.6%) and unique deduplicated mapping rate (53.9%) (fig. S1C), although it used more cfDNA input (from 5 ml of plasma). This highlights the advantage of cfTAPS to generate higher-quality and more complex data than cfDNA WGBS while requiring less cfDNA input.

Subsequently, we assessed accuracy of cfTAPS to detect 5mC based on spike-in controls that have modified and unmodified cytosines in the known positions. We used CpG-methylated lambda DNA to estimate the conversion of 5mC. Two samples had a low conversion rate below 85% and were excluded from downstream analysis (table S1). The remaining 85 samples had a mean 5mC conversion rate of 97.0% or a false-negative rate (nonconversion rate of 5mC) of 3.0% (Fig. 1C). The false-positive rate (conversion rate of unmodified C), estimated on the basis of unmodified amplicon spike-in, was 0.28%, which confirms that cfTAPS allows highly sensitive and specific detection of 5mC in cfDNA (Fig. 1C). We further confirmed high reproducibility of cfTAPS between technical replicates (fig. S1D).

### Whole-genome DNA methylation from cfTAPS

**Fig. 2.**
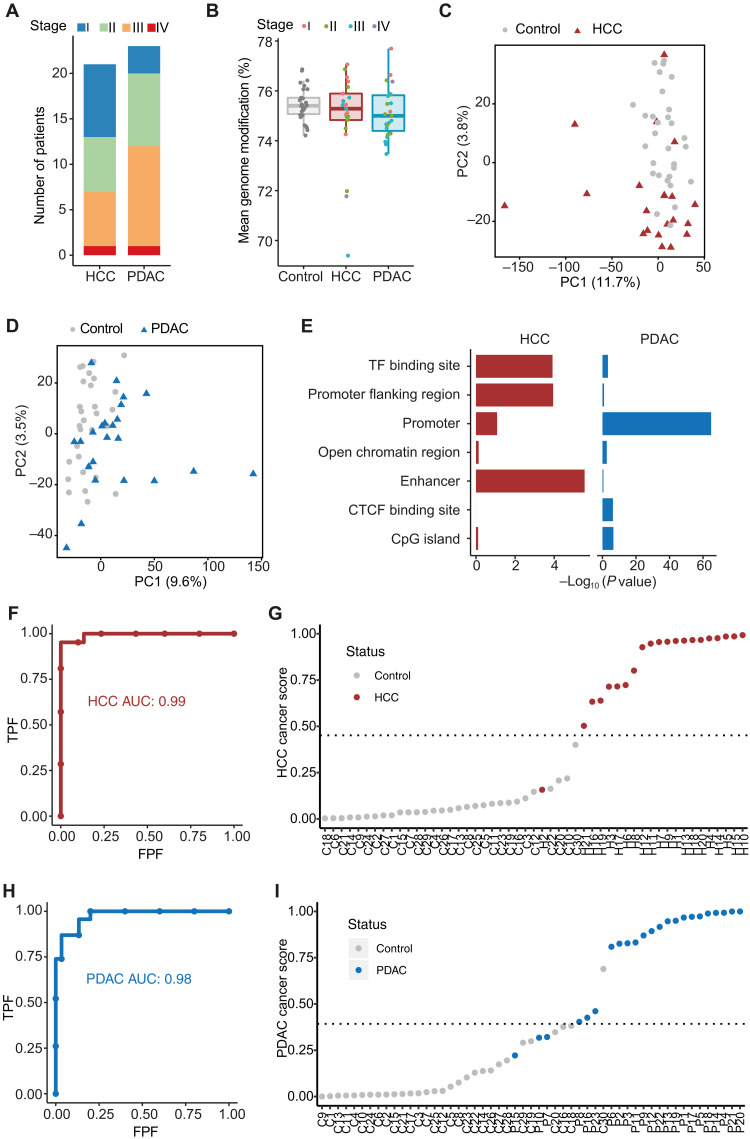
cfDNA methylation in clinical samples. (**A**) Cancer stage distribution of 21 HCC and 23 PDAC patients included in the study. (**B**) Mean per CpG genome modification level in noncancer controls, HCC, and PDAC cfDNA. Each dot represents an individual sample. (**C**) PCA plot of cfDNA methylation in 1-kb genomic windows in noncancer controls and HCC. (**D**) PCA plots of cfDNA methylation in 1-kb genomic windows in noncancer controls and PDAC. (**E**) The overrepresentation analysis on the regions correlated most with PC2 for HCC and PC1 for PDAC in regulatory regions. (**F**) ROC curve of model classification performance based on differentially methylated enhancers in HCC and noncancer controls (*n* = 51, HCC = 21, noncancer controls = 30). FPF, false positive fraction; TPF, true positive fraction. (**G**) LOO cancer prediction scores for HCC and noncancer controls. Dashed line represents probability score threshold. Samples with a probability score above this threshold were predicted as HCC. (**H**) ROC curve of model classification performance based on differentially methylated promoters between PDAC and noncancer controls (*n* = 53, PDAC = 23, noncancer controls = 30). (**I**) LOO cancer prediction scores for PDAC and noncancer controls. Dashed line represents probability score threshold. Samples with a probability score above this threshold were predicted as PDAC.

Next, we sought to characterize the cfDNA methylome in the 85 cfDNA samples that passed initial quality control. Our cohort included samples from 21 patients with HCC, 23 with PDAC, 30 noncancer controls, 4 patients with cirrhosis, and 7 with pancreatitis (fig. S2A). Cirrhosis and pancreatitis are precancerous conditions affecting the liver and pancreas, respectively (*25*, *26*). Most PDAC and HCC patients in our cohort were at a nonmetastatic stage, with 52% of PDAC and 67% HCC patients at stages I and II (Fig. 2A and table S2). Among the 21 HCC patients, only 4 (19%) had elevated levels of AFP (over 20 ng/ml; table S2) (*22*). Among the 18 PDAC patients who had CA19-9 measurement, 16 (89%) had elevated levels of CA19-9 (over 37 U/ml; table S2) (*27*). However, CA19-9 level is often elevated in nonmalignant conditions including inflammatory disease (*23*). Of note, our noncancer controls were collected from an endoscopy clinic and were enriched with gastrointestinal inflammatory conditions such as Crohn’s disease and colitis (table S2). While distinguishing these noncancer controls from cancer patients is more challenging than a typically healthy control group, this may provide a more real-world comparison of a diagnostic test in an aging population.

We first analyzed global methylation levels of cfDNA in cancer and control samples. CfDNA methylation displayed a typical bimodal distribution in all groups, with most CpG sites either fully methylated or unmethylated (fig. S2B). Average CpG methylation level in control samples was 75.5% and was similar in cancer cfDNA (HCC, 74.9%; PDAC, 75.1%). Previously reported global cfDNA hypomethylation in HCC (*10*) was only observed in a few samples with late stage or large tumor size (Fig. 2B and fig. S2, C to F). By contrast, we observed a higher variance of methylation in 1-Mb genomic windows between cancer patients and controls (fig. S2, G and H).

To investigate whether whole-genome cfDNA methylation signatures have the potential to discriminate between cancer patients and noncancer controls, we first performed principal components analysis (PCA) of cfDNA methylation in 1-kb genomic windows. Both HCC (Fig. 2C) and PDAC samples (Fig. 2D) showed partial separation from controls in principal component 2 (PC2) and PC1, respectively. Note that the inflammatory patients (Crohn’s disease and colitis) do not separate from the other noncancer controls (fig. S2I). We then investigated where the windows that most contributed to the cancer/control separation were enriched in the genome. We found that the top 200 windows with the highest correlation with PC2 for HCC were enriched in enhancers (see Materials and Methods; Fig. 2E). Conversely, the 200 windows most highly correlated with PC1 for PDAC were highly enriched in promoters (Fig. 2E), suggesting that different cancer types have different cfDNA methylation signals.

### Differential DNA methylation from cfTAPS

Because methylation patterns in regulatory regions substantially contributed to discrimination between cancer and controls in unsupervised analysis, we investigated the predictive potential of cfDNA methylation in enhancer and promoter regions for HCC and PDAC prediction, respectively, using a supervised machine learning approach with leave-one-out (LOO) cross-validation. Briefly, in each round of LOO cross-validation, we used one sample as a validation set and the remaining samples for model training. Within each fold, we identified differentially methylated enhancers and promoters for HCC and PDAC, respectively, and used them to train a regularized generalized linear model classifier (glmnet) (*28*) to distinguish each cancer type from the control samples. This model was then evaluated on the held-out test sample for each fold (fig. S3A). Cirrhosis and pancreatitis samples were not included in model building but were used as an independent validation set to evaluate performance of our classifiers to discriminate between cancer and premalignant conditions.

We achieved excellent prediction of HCC [AUC (area under the curve) = 0.99] based on differentially methylated enhancers (see Materials and Methods; Fig. 2, F and G, and table S3). Moreover, on the basis of predicted scores, three of four cirrhosis samples could be distinguished from HCC, suggesting that our model is able to detect cancer-specific features (fig. S3B). We then performed gene ontology (GO) analysis on the differentially methylated enhancers and found significant enrichment in signaling pathways commonly affected in liver cancer, including regulation of RAC1 activity (*29*) and interleukin-8 (IL-8)– and CXCR1-mediated signaling (fig. S3C) (*30*). For example, in cfDNA of HCC patients, we observed significant hypermethylation of the enhancer that regulates expression of the *DLC1* gene, a tumor suppressor for human liver cancer involved in RAC1 and Rho signaling pathways (fig. S3D) (*31*).

We achieved accurate prediction of PDAC (AUC = 0.98) based on differentially methylated promoters (see Materials and Methods; Fig. 2, H and I, and table S4). Similarly, the classifier was able to predict six of seven pancreatitis samples as noncancer despite not being trained on any pancreatitis samples (fig. S3E). Differentially methylated promoters in PDAC cfDNA were enriched in signaling pathways affected in PDAC, including RB1 regulation (*32*) and p38 signaling pathways (fig. S3F) (*33*). For instance, we found significant hypermethylation in the *RB1* gene promoter (fig. S3G), a well-studied tumor suppressor gene. Hypermethylation of *RB1* promoter was previously found in human cancers (*34*), and down-regulation (*35*) of RB1 was reported in pancreatic cancer.

Last, we validated our HCC model on an independent dataset from a recent cfDNA WGBS study, which contains four HCC patients and four noncancer controls (*24*). We found that our models, built on differentially methylated enhancers identified from cfTAPS data, were able to correctly classify all HCC and noncancer controls from this external dataset (fig. S3H). The high sequencing depth of cfTAPS is essential for de novo differential methylation analysis from cfDNA (*36*), and the differentially methylated regions (DMRs) identified were significantly decreased when we down-sampled our data to 100 to 200 million read pairs (fig. S3I). Together, cfTAPS enables whole-genome discovery of DMRs in cfDNA, and the distinct methylation patterns in regulatory regions enable accurate prediction of HCC and PDAC.

### cfTAPS informs tissue of origin

CfDNA methylation has been shown to provide tissue-of-origin information (*8*, *9*, *11*–*14*). Most approaches use 450K methylation array tissue data (*9*, *13*), which covers less than 1% of CpGs in the human genome, to infer tissue contribution from cfDNA methylation. To further use the whole-genome information from cfTAPS for cfDNA deconvolution (*11*, *14*), we collated CpG-level methylation data from 144 publicly available tissue and blood cell WGBS, stratified into 32 physiologically distinct tissue and blood cell types, including liver tumor tissue (table S5). Given the prevalence of tissue-specific DNA methylation in enhancer regions (*37*), we constructed an enhancer-aggregated reference map of tissue methylation (see Materials and Methods). The resulting methylation reference map displays good clustering of blood and immune cell types and even physiologically related solid tissues (fig. S4A).

**Fig. 3.**
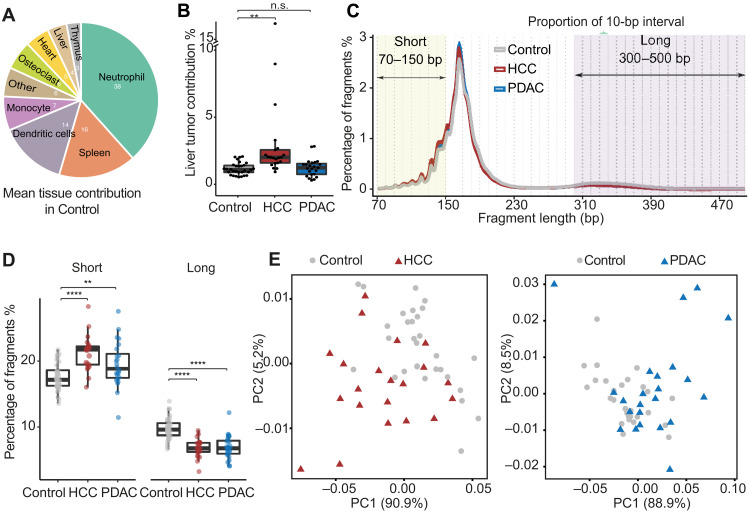
cfTAPS enables analysis of tissue of origin and fragmentation patterns in cfDNA. (**A**) Mean tissue contribution in noncancer individuals estimated by NNLS. Tissue contributions less than 1.5% are aggregated as “Other.” (**B**) Boxplot showing the estimated liver cancer contribution within noncancer, HCC, and PDAC groups. Statistical significance was assessed with a paired *t* test. n.s., not significant. (**C**) Length distribution of cfDNA fragments in the three groups. For each sample, proportion in 10-bp intervals of long cfDNA fragments (300 to 500 bp) was used as fragmentation features for PCA and machine learning. (**D**) Boxplot showing proportion of short (70 to 150 bp) and long (300 to 500 bp) fragments in noncancer controls, PDAC, and HCC. The Kruskal-Wallis test was performed to test differences in fragment size distribution between groups. Statistically significant differences are marked with asterisks (**P < 0.01, ****P < 0.0001). (**E**) PCA plot of cfDNA 10-bp fragment fraction in noncancer controls and HCC (left) and noncancer controls and PDAC (right).

We calculated tissue contribution in cfTAPS samples by performing nonnegative least squares regression (NNLS) (*13*, *14*). cfDNA tissue contribution was broadly similar between cancer and control groups, in agreement with previous reports (*13*, *14*), with blood and immune cells dominant, and lower proportions of solid tissues (Fig. 3A, fig. S4B, and table S6). We observed a significantly increased liver tumor contribution in HCC alone (paired *t* test, *P* = 0.0016; Fig. 3B) and a significantly increased memory T cell contribution in PDAC samples (paired *t* test, *P* = 0.028; fig. S4C). We trained a regularized generalized linear model based on tissue contribution, evaluating all samples using LOO cross-validation, and showed that it can correctly separate most samples in both cancer types (HCC versus noncancer control, AUC = 0.77; PDAC versus noncancer control, AUC = 0.81). However, these models perform worse at distinguishing pancreatitis and cirrhosis compared to methylation-based models (fig. S4, D to I). Tissue deconvolution is currently limited by the availability of public WGBS data. Nevertheless, these results indicate that cfTAPS provides valuable tissue-of-origin information for early cancer detection.

### Fragmentation patterns from cfTAPS

Although the main purpose of cfTAPS is DNA methylation sequencing, it only induces base changes at modified cytosines, thus keeping most of the DNA intact. We can therefore extract additional genetic information from cfTAPS data to further improve the sensitivity of early cancer detection. We first investigated CNVs from cfTAPS data. As expected with our nonadvanced cancer cohort, we only predicted CNVs in four HCC and three PDAC patients (fig. S5, A and B). Next, we investigated whether cfTAPS can retain reliable cfDNA fragmentation information, which has recently been shown to change substantially during cancer development and has therefore been adopted in cancer detection assays (*38*, *39*).

We first confirmed that cfDNA fragmentation patterns detected with cfTAPS are concordant with cfDNA fragmentation pattern generated by whole-genome sequencing (WGS) (*38*), with the dominant peak at 167 base pairs (bp), a secondary peak at ~320 bp, and smaller peaks below 167 bp with 10-bp periodicity, reflecting nucleosomal fragmentation patterns (Fig. 3C and table S7). By contrast, fragmentation patterns were clearly different in previously published cfDNA WGBS (*24*), as the 10-bp oscillations in the cfDNA fragmentation profile were lost presumably because of DNA damage (fig. S6A). Consistent with previous cfDNA WGS (*38*), we found that cancer patients have a higher frequency of cfDNA fragments below 150 bp (Kruskal-Wallis test: HCC, *P* = 6.871 × 10^−6^; PDAC, *P* = 0.006731) and a lower proportion of long fragments between 310 and 500 bp (Kruskal-Wallis test: HCC, *P* = 2.627 × 10^−7^; PDAC, *P* = 1.263 × 10^−6^) compared to noncancer controls (Fig. 3D), further confirming the faithful preservation of cfDNA fragmentation information in cfTAPS.

We then developed a new approach for characterization of cfDNA fragmentation profiles using cfTAPS. Briefly, we divided the cfDNA fragmentation distribution into 10-bp bins and calculated the proportion of fragments in each 10-bp bin (Fig. 3C). We found that cfDNA long fragment (300 to 500 bp) length proportion in 10-bp bins separated PDAC and HCC from controls in PCA (Fig. 3E). We further showed that this cfDNA fragmentation signature can be used to distinguish HCC and PDAC from noncancer controls with high accuracy (HCC, AUC = 0.92; PDAC, AUC = 0.84) (fig. S6, B, C, E, and F). However, this approach was less accurate at distinguishing cancer from cirrhosis and pancreatitis compared to methylation-based classifiers (fig. S6, D and G), suggesting that fragmentation information is less cancer specific.

### Multi-cancer detection with cfTAPS

**Fig. 4.**
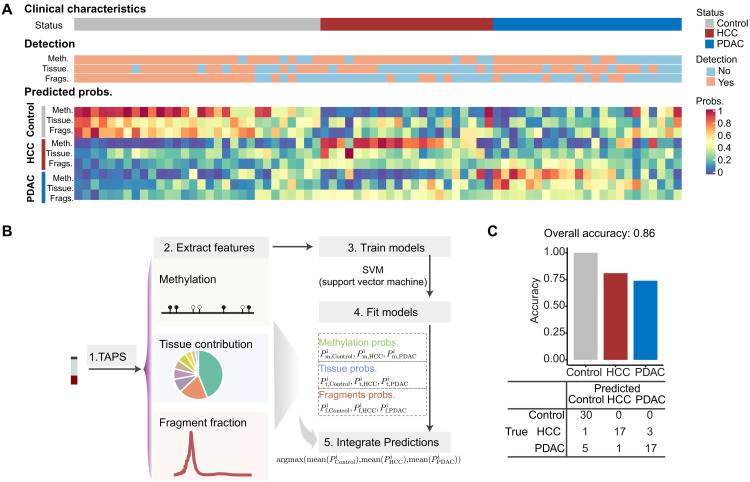
Integrating multimodal features from cfTAPS enhances multicancer detection. (**A**) Heatmap showing individual model performance on multicancer prediction and the predicted probabilities for each patient. Each vertical column is a patient. Detection yes/no means that patients are correctly classified or misclassified on the basis of a particular feature. Predicted score means the probability of classifying the patients to a specific group based on a particular feature. (**B**) Schematic detailing the method of integrating multiple features (DNA methylation, tissue contribution, and fragmentation fraction) extracted from cfTAPS data for multicancer prediction. (**C**) Actual and predicted patient status calculated in LOO cross-validation.

We next investigated the utility of cfTAPS for multicancer detection. We selected top five DMRs of each pairwise comparison (noncancer controls versus HCC, noncancer controls versus PDAC, and HCC versus PDAC; see Materials and Methods) as features in the multicancer differential methylation model. We trained a support vector machine (SVM) model to estimate the respective probability that the blood sample came from each group. We built similar models using tissue contribution and fragmentation profile. Using LOO cross-validation, we found that the methylation model can achieve an overall accuracy of 0.77, which outperforms the tissue contribution model and fragmentation profile model (accuracy of 0.62 and 0.46, respectively; Fig. 4A and fig. S7A).

To further enhance the multicancer predictive model, we built a multimodal classifier that combined differential methylation, tissue contribution, and fragment profile (Fig. 4B). This integrated model took the averaged scores across the three modalities and used the most confident prediction for each sample. The overall accuracy of the combined model was 0.86 (64 of 74 were classified correctly), and the accuracy for distinguishing controls from any cancer type is 0.92 (Fig. 4C), which highlights the benefits of incorporating multimodal information for cancer type prediction. Last, we explored the DMRs used for multicancer prediction (fig. S7B and table S8). We found that the nearby genes of these regions were enriched in Notch and Wnt signaling (*40*) and epidermal growth factor receptor (ErbB) signaling (*41*), which provides biological support for these potential multicancer biomarkers (fig. S7C).

## DISCUSSION

In this study, we successfully optimized and applied cfTAPS to characterize whole-genome base-resolution methylome in cfDNA from HCC, PDAC, and noncancer controls. Using only 10 ng of cfDNA, cfTAPS libraries demonstrated greatly improved sequencing quality and depth compared to previous cfDNA WGBS. Using less cfDNA input than previous studies, cfDNA TAPS generated the most comprehensive cell-free methylation to date. The much higher yield of informative reads allows cfTAPS to extract more information from a given amount of cfDNA and makes it a viable option for large-scale cfDNA methylation studies.

The deep sequencing achieved by cfTAPS enables detailed analysis of the cell-free methylome and whole-genome discovery of methylation biomarkers for early cancer detection. While we do not observe significant global hypomethylation, suggesting that in our cohort the fraction of cfDNA derived from tumor cells is low (as corroborated by the lack of CNVs in most cancer patients included in the study), we found that local methylation signals in regulatory regions such as enhancers and promoters contained cancer-specific information that could accurately distinguish HCC and PDAC from controls. This is particularly promising considering the inflammation-enriched real-world control group used in our patient cohort and that our HCC model can correctly identify all HCC and control patients from a cfDNA WGBS dataset as an independent validation (*24*).

Another important advantage of cfDNA methylation for early cancer detection is the ability to determine tissue-of-origin information. Using currently available public WGBS tissue databases, we performed a whole-genome tissue deconvolution of cfTAPS data and observed increased liver tumor contribution in HCC cfDNA and distinct immune signatures in cancer cfDNA. The tissue deconvolution itself can be used for cancer detection. Last, because TAPS converts modified cytosine directly, it maximally retains the underlying genetic information compared to other approaches that convert unmodified cytosines. In this study, we extracted CNVs and fragmentation information from cfTAPS, the latter of which is lost in cfDNA WGBS. We further demonstrated that an integrated approach combining differential methylation, tissue of origin, and fragmentation profiles could improve the model performance for multicancer detection.

Despite promising results, our study has marked limitations. First, our approach was tested on a relatively low number of patients. We were able to validate our methylation-based HCC classifier on an independent cfDNA HCC WGBS dataset. However, because of limited sample size and lack of available public cfDNA PDAC whole-genome methylation data, findings of this study have yet to be validated on a bigger, independent cohort, including the performance comparison to AFP and CA19-9 (AFP and CA19-9 measurements were not available for the noncancer controls in our study). Second, because of the limited amount of publicly available genome-wide tissue methylation data (for instance, no PDAC tissue data were available) and the fact that we are comparing TAPS data to a WGBS database, the current investigation was far from the full potential of tissue-of-origin information from cfTAPS (*42*). In the future, a comprehensive cell type–specific TAPS reference database could improve the resolution and sensitivity of tissue deconvolution from cfTAPS considerably. We note that emerging novel computational approaches, such as those that use read-level methylation information (*24*), will also aid future methylation deconvolution efforts. Third, our predictive pipeline uses simple, out-the-box classification models to demonstrate the signal present in cfTAPS data; as we collect more data, future work will explore more sophisticated machine learning models that allow automated feature extraction from genome-wide methylomes. cfTAPS should retain various other genetic and epigenetic information such as microbiome (*14*) and nucleosome positioning (*43*), which are interesting avenues to explore in subsequent work. Future studies with the proper design (i.e., germline controls) would also allow simultaneous detection of mutations from cfTAPS in either a whole-genome or targeted sequencing manner. These additional modalities from a single cfTAPS assay would further improve sensitivity and ability to detect outliers within highly heterogeneous disease cohorts (*18*).

In this first proof-of-concept study, we conducted deep whole-genome methylome sequencing with cfTAPS, which is necessary for unbiased methylation marker discovery at the whole-genome level. These potential methylation markers can be used in targeted cfTAPS for future large-scale validation studies to reduce the sequencing cost. On the other hand, we showed that whole-genome cfTAPS has the benefit of offering a wealth of information such as tissue-of-origin and fragmentation profiles. Recently, this type of breadth-over-depth approach has been demonstrated in whole-genome cfDNA mutational sequencing in the minimal residual disease setting (*3*, *4*). While the cost of sequencing continues to drop, it remains to be tested whether whole-genome or targeted DNA methylation sequencing is the best approach for early cancer detection.

## MATERIALS AND METHODS

### Experimental design

Whole-blood samples from 30 noncancer controls were obtained from John Radcliffe Hospital (ethical approvals IDs 16/YH/0247 and 18/WM/0237). Pancreatitis blood samples from eight patients were obtained from John Radcliffe Hospital. The study was approved by Oxfordshire Research Ethics Committee A (REC-A) (10/H0604/51) and is registered on the U.K. National Institute for Health Research (NIHR) portfolio as study number 10776. PDAC patients were consented for this study via the Oxford Radcliffe Biobank (09/H0606/5+5, project: 19/A177), and whole-blood samples were collected from 24 patients. Collection of plasma samples from 21 HCC and 4 cirrhosis patients was REC-approved [ethical approval 2/NE/0395, IRAS (Integrated Research Application System) project ID: 116370]. No sample size calculations were performed. Sample size was determined on the basis of availability. PDAC, HCC, pancreatitis, and cirrhosis samples were collected from subjects with clinically diagnosed disease. Noncancer control samples were collected from individuals without cancer diagnosis at the time of sample collection or previous history of cancer.

The main goal of the study was comprehensive, multidimensional characterization of cfDNA in cancer and controls by whole-genome methylation sequencing using TAPS. CfDNA TAPS libraries were constructed and paired-end 150 bp–sequenced on a NovaSeq 6000 sequencer (Illumina). Technical details are described in the sections below. Samples with 5mC conversion below 90% calculated on the basis of methylated lambda spike-in control were excluded from downstream analysis.

### Collection and preparation of cfDNA samples

Blood was collected into EDTA-coated Vacutainers. Plasma was separated from collected blood samples within 4 hours from collection. Plasma was collected by centrifuging blood at 1600*g* for 10 min at 4°C and 16,000*g* for 10 min at 4°C and stored at −80°C for cfDNA purification. cfDNA from plasma was extracted using the QIAamp Circulating Nucleic Acid Kit (Qiagen). cfDNA was quantified with Qubit Fluorometer (Life Technologies).

### Preparation of carrier DNA and spike-in controls

Carrier DNA was prepared by PCR amplification of the pNIC28-Bsa4 plasmid (Addgene, catalog no. 26103) in a reaction containing 1 ng of DNA template, 0.5 μM primers (forward: 5′-AGGCAACTTTATGCCCATGCAA-3′, reverse: 5′-CCAAGGGGTTATGCTAGTTATTGC-3′), and 1× Phusion High-Fidelity PCR Master Mix with HF Buffer (Thermo Fisher Scientific). The CpG-methylated lambda DNA and 2-kb unmodified spike-in control DNA were prepared as described previously (*17*). CpG-methylated lambda DNA, carrier DNA, and 2-kb unmodified control were fragmented by Covaris M220 (peak incident power, 50 W; duty factor, 20%; cycles per burst, 200; time, 150 s) and size-selected on 0.9 to 1.2× AMPure XP beads to select for 150- to 250-bp fragments.

### Preparation of sequencing adapters

Adapter oligos (5′-ACACTCTTTCCCTACACGACGCTCTTCCGATCT-3′, 5′-/5Phos/GATCGGAAGAGCACACGTCT-3′) were obtained from (Integrated DNA Technologies) with high-performance liquid chromatography purification. Adapter oligos were annealed together in a 50-μl reaction containing 15 μM each oligo, 10 mM tris-Cl (pH 8.0), 0.1 mM EDTA (pH 8.0), and 50 mM NaCl with the following program: 2 min at 95°C, 140 cycles of 20 s at 95°C (decrease temperature 0.5°C every cycle), and hold at 4°C. Annealed 15 μM Illumina multiplexing adapters were then aliquoted into small single-use vials and stored at −80°C.

### mTet1CD oxidation

mTet1CD was prepared as described previously (*17*). DNA was incubated in a 50-μl reaction containing 50 mM Hepes buffer (pH 8.0), 100 μM ammonium iron (II) sulfate, 1 mM α-ketoglutarate, 2 mM ascorbic acid, 2 mM dithiothreitol, 100 mM NaCl, 1.2 mM adenosine triphosphate, and 4 μM mTet1CD for 80 min at 37°C. After that, 0.8 U of proteinase K (New England Biolabs) was added to the reaction mixture and incubated for 1 hour at 50°C. The product was cleaned up on Bio-Spin P-30 Gel Column (Bio-Rad) and 1.8× AMPure XP beads following the manufacturer’s instruction.

### PyBr reduction

Oxidized DNA in 35 μl of water was reduced in a 50-μl reaction containing 600 mM sodium acetate solution (pH 4.3) and 1 M PyBr (Alfa Aesar) for 16 hours at 37°C and 850 rpm in the Eppendorf ThermoMixer. The product was purified using Zymo-Spin columns.

### cfDNA TAPS

cfDNA (10 ng) was spiked-in with 0.15% CpG-methylated lambda DNA and 0.015% unmodified 2-kb control and used for an end-repair and A-tailing reaction and ligated to Illumina Multiplexing adapters with a KAPA HyperPrep kit according to the manufacturer’s protocol. Subsequently, 100 ng of carrier DNA was added to ligated libraries and samples were double-oxidized with mTet1CD and reduced with PyBr as described above. Converted libraries were amplified using NEBNext Multiplex Oligos for Illumina (96 Unique Dual Index Primer Pairs) with KAPA Hifi Uracil Plus Polymerase for seven cycles and cleaned up on 1× AMPure XP beads. CfDNA TAPS libraries were paired-end 150 bp–sequenced on a NovaSeq 6000 sequencer (Illumina).

### TAPS mapping and preprocessing

Raw sequenced reads were processed with trim:galore (version 0.6.2; https://bioinformatics.babraham.ac.uk/projects/trim:galore/) to trim adapter and low-quality bases with the following parameters: --paired --length 35 --gzip --cores 2. Clean reads were aligned to human reference genome (GRCh38, ftp://ftp.ncbi.nlm.nih.gov/genomes/all/GCA/000/001/405/GCA_000001405.15_GRCh38/seqs_for_alignment_pipelines.ucsc_ids/GCA_000001405.15_GRCh38_no_alt_analysis_set.fna.gz.) combining spike-in sequences using bwa mem (*44*) (version 0.7.17-r1188) with the following parameters: -I 500,120,1000,20. Reads with MAPping Quality Value (MAPQ) <1 were excluded from further analysis. Picard MarkDuplicates (version 2.18.29-SNAPSHOT) was used to identify duplicate reads. MethylDackel extract (version 0.5.0; https://github.com/dpryan79/MethylDackel) was used for methylation calling using the following parameters: -q 10 -p 13 -t 4 --mergeContext --OT 10,140,75,75 --OB 10,140,75,75. CpG sites overlapped with common single-nucleotide polymorphism (SNP) (dbSNP153) (*45*), blacklisted regions (*46*), centromeres (*45*), and sex chromosomes were excluded for further analysis.

### cfDNA WGBS analysis

CfDNA WGBS data were downloaded from EGAD00001004317 (*24*). Raw sequenced reads were processed with trim:galore (version 0.6.2; https://bioinformatics.babraham.ac.uk/projects/trim:galore/): We trimmed adapter and low-quality bases with the following parameters: --paired --length 35 --gzip --cores 2. Clean reads were aligned to human reference genome (GRCh38) using bismark (*47*) (Bismark version v0.22.0) with default parameters. deduplicate_bismark was used for deduplication. Samtools (*48*) was used to filter the fragments with -q 10, and only reads mapped in proper pairs were used for fragmentation analysis. Then, bismark_methylation_extractor was used to extract methylation from deduplicated bam files with default parameters.

### PCA on DNA methylation and feature overrepresentation analysis

The genome was binned into 1-kb windows. Methylation level was calculated using the number of methylated CpGs divided by the number of total CpGs sequenced. Windows with mean CpG coverage (number of total CpG sequenced/total number of CpG positions) < 2 were excluded for further analysis. Dimdesc (*49*) was used with parameter proba = 0.01 to determine the regions that contribute most to each principal component obtained by the PCA function (largest eigenvalues of each eigenvector). Bedtools (*50*) fisher was used to test the number of overlaps between the top 200 contributing regions (sorted by absolute correlation value) and the selected genomic features. Selected genomic features included regulatory element (*51*) from Ensemble (ftp://ftp.ensembl.org/pub/release-97/regulation/homo_sapiens/homo_sapiens.GRCh38.Regulatory_Build.regulatory_features.20190329.gff.gz) and CpG islands from UCSC (http://hgdownload.soe.ucsc.edu/goldenPath/hg38/database/cpgIslandExt.txt.gz).

### Two-class prediction using DNA methylation signature

Two-class prediction models were trained and evaluated on the basis of a LOO approach. Briefly, one sample was held out as the testing set, while the remaining samples were used for model training. DMRs (promoters for PDAC and enhancers for HCC) were identified in the training set by *t* test (*P* < 0.002, methylation difference > 0.05). In each LOO fold, 443 to 775 differentially methylated enhancers and 160 to 318 differentially methylated promoters were identified in the HCC versus noncancer control and PDAC versus noncancer control feature selection steps, respectively. In total, 1521 enhancers and 531 promoters were selected during the cross-validation process. The predictive model was built on selected DMRs using cv.glmnet (*28*) and validated on the test sample. This procedure was repeated *N* times, where *N* = number of samples. Receiver operating characteristic (ROC) curves were prepared in R based on the predicted scores of held-out test samples from cvglm models. Cirrhosis patients and cfDNA WGBS data (*24*) were used as independent validation sets to evaluate the performance of HCC model. Pancreatitis patients were used as independent validation set to evaluate the performance of PDAC model. Aligned Binary Alignment Map (BAM) files were down-sampled from 100 million to 200 million read pairs using samtools view (*48*). For each down-sampled set, we used the method described above to detect DMRs. Ref DMRs were defined as the total unique DMR in the LOO cross-validations. The percentage of ref DMRs was computed by dividing the overlapped DMR between the down-sampled set and the ref DMR and the total ref DMR.

### GO analysis of DMRs

Genes regulated by differentially methylated enhancers in HCC cfDNA were identified using the GeneHancer database (*52*). The genes closest to the differentially methylated promoters in PDAC were identified as related using following R packages: AnnotationHub (version 2.18.0), TxDb.Hsapiens.UCSC.hg38.knownGene (version 3.10.0), and org.Hs.eg.db (version 3.10.0). GO analysis was performed on these identified genes using Enrichr tool (*53*) against National Cancer Institute–Nature Pathway Interaction database.

### Tissue reference map

CpG-level tissue methylation data were collated from six public sources (table S5). After filtering diseased, sex-specific, and low-coverage samples, we retained 144 healthy, adult tissue samples, grouped into 32 physiologically distinct tissue groups (table S6). One hundred thirty-three of 144 samples were already aligned to hg38; the remaining 11 samples were converted from hg19 to hg38 using the UCSC hgLiftOver tool (*54*).

We filtered 79,000 enhancers from Ensembl Regulatory Build (*51*) using a tissue-specific DMR finding algorithm similar to Moss *et al.* (*13*). Specifically, this algorithm performs pairwise one-versus-all comparisons for each tissue group in the reference atlas, selecting the regions that show the largest median methylation difference and consistent methylation across the tissue group in question. As in Moss *et al.* (*13*), we also calculated pairwise tissue group correlations and included DMRs that best separated each tissue group from the first and second most highly correlated tissue.

### Tissue deconvolution by NNLS regression

Tissue deconvolution was performed using NNLS and implemented using Scipy’s optimize function (*55*) in Python 3.8. Given a tissue reference matrix *A* and a vector of observed methylation ratios *y_s_* in a sample *s*, we estimate the tissue contribution *x* by solving the following minimization problem

$$\text{min}{\left\|\mathrm{Ax}-y_{s}\right\|}^{2}$$

subject to *x* ≥ 0

### Fragmentation analysis

The length of the DNA fragments was obtained from alignment files using samtools (*48*). Fragmentation profiles were calculated as the fraction of cfDNA fragments at 10-bp length range bins. PCA and plots were generated in R.

For fragmentation-based prediction, the proportion of cfDNA fragments (300 to 500 bp) in 10-bp length range bins was calculated. Models were built and trained by LOO approach using cv.glmnet (*28*) method. ROC curves were prepared in R based on prediction scores from validation.

### CNV analysis

Alignment files for each sample were down-sampled to 225 million read pairs with samtools (*48*) view. QDNAseq (*56*) package was used for CNV analysis. The bin annotation was downloaded from QDNAseq.hg38 (https://github.com/asntech/QDNAseq.hg38), and bin size 100 kb was used. Regions that were blacklisted or have mappability less than 80 were excluded for further analysis. Cutoffs 0.8 and 1.2 were used to define copy number losses and gains, respectively, in the callBins function. Patients that have copy number aberrations with length range bigger than 500 kb were classified as patients with CNV.

### Three-class prediction models

Three-class prediction models were trained and evaluated on the basis of a LOO approach. For DNA methylation, we initially narrow down the candidate features to 824,320 1-kb windows encompassing mapping to regulatory regions as mentioned previously. The methylation model aims to capture the cancer type–specific methylation change by selecting DMRs based on a pairwise comparison using a *t* test. DMRs were then ranked by *P* value, and the top five DMRs in each pairwise comparison were selected for model training. The prediction model was built on DMRs selected among the training sets using an SVM model implemented in the caret package (*57*) (train method = “svmLinear2”) and validated on the test sample. This procedure was repeated *N* times, where *N* = number of samples. For tissue contribution and fragmentation fraction, the raw matrices were used to build models following the same method as for DMRs. These three models were integrated by taking the average (mean) predictions across the three modalities, where the selected prediction in each case was the one with the maximum average predicted score.

## Notes

View/request a protocol for this paper from *Bio-protocol*.
