# Supplementary material for: Cell-free DNA TAPS provides multimodal information for early cancer detection
Source: Sci Adv. 2021 Sep 1;7(36):eabh0534. doi: 10.1126/sciadv.abh0534 (PMC8442905; doi:10.1126/sciadv.abh0534)
Supplement: Supplementary file 1 — Figs. S1 to S7 Legends for tables S1 to S8 [file sciadv.abh0534_sm.pdf]

Science Advances
AAAS

Supplementary Materials for

Cell-free DNA TAPS provides multimodal information for early cancer detection

Paulina Siejka-Zielińska, Jingfei Cheng, Felix Jackson, Yibin Liu, Zahir Soonawalla, Srikanth Reddy, Michael Silva, Luminita Puta, Misti Vanette McCain, Emma L. Culver, Noor Bekkali, Benjamin Schuster-Böckler, Pier Francesco Palamara, Derek Mann, Helen Reeves, Eleanor Barnes, Shivan Sivakumar*, Chun-Xiao Song*

*Corresponding author. Email: chunxiao.song@ludwig.ox.ac.uk (C.-X.S.); shivan.sivakumar@oncology.ox.ac.uk (S.S.)

Published 1 September 2021, *Sci. Adv.* **7**, eabh0534 (2021)
DOI: 10.1126/sciadv.abh0534

The PDF file includes:

Figs. S1 to S7
Legends for tables S1 to S8

Other Supplementary Material for this manuscript includes the following:

Tables S1 to S8

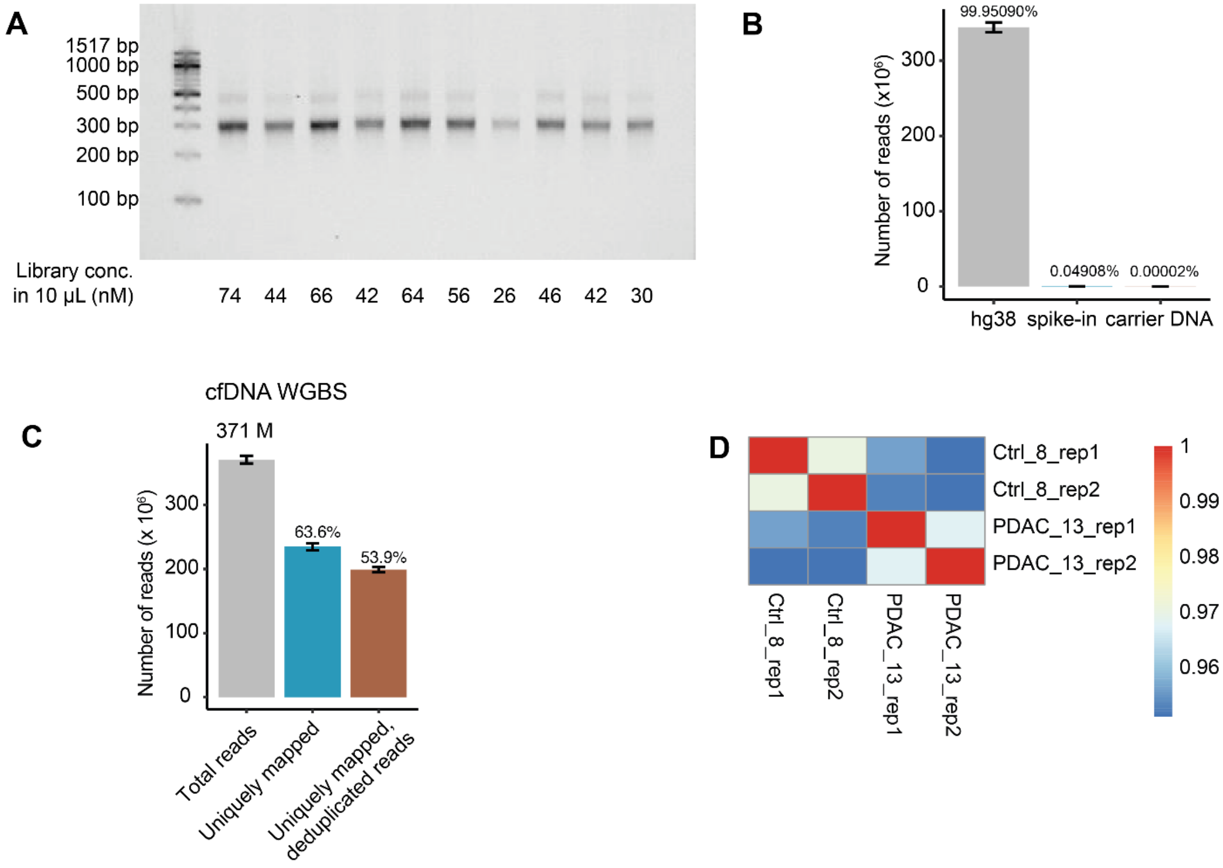

Fig. S1. cfDNA TAPS.

(A) Agarose gel of 10 representative cfDNA TAPS libraries after post-amplification clean-up. All cfDNA TAPS libraries were prepared from 10 ng of cfDNA and amplified for 7 PCR cycles. **(B)** Number of mapped read-pairs for hg38, spike-ins and carrier DNA in 87 cfDNA TAPS libraries. Mean percentage of mapped read-pairs compared to total read-pairs is shown above the bars. Error bars represent standard error. **(C)** Number of total reads, uniquely mapped reads and uniquely mapped, PCR deduplicated reads in cfDNA WGBS (EGAD00001004317) *(24)*. Total number of reads and mean percentage of uniquely mapped reads and deduplicated reads compared to the total reads are shown above the bars. Error bars represent standard error. **(D)** Correlation between technical replicates of cfDNA TAPS libraries prepared from the same cfDNA samples sequenced to low depth 2.6×. Methylation was calculated in 100 kb windows.

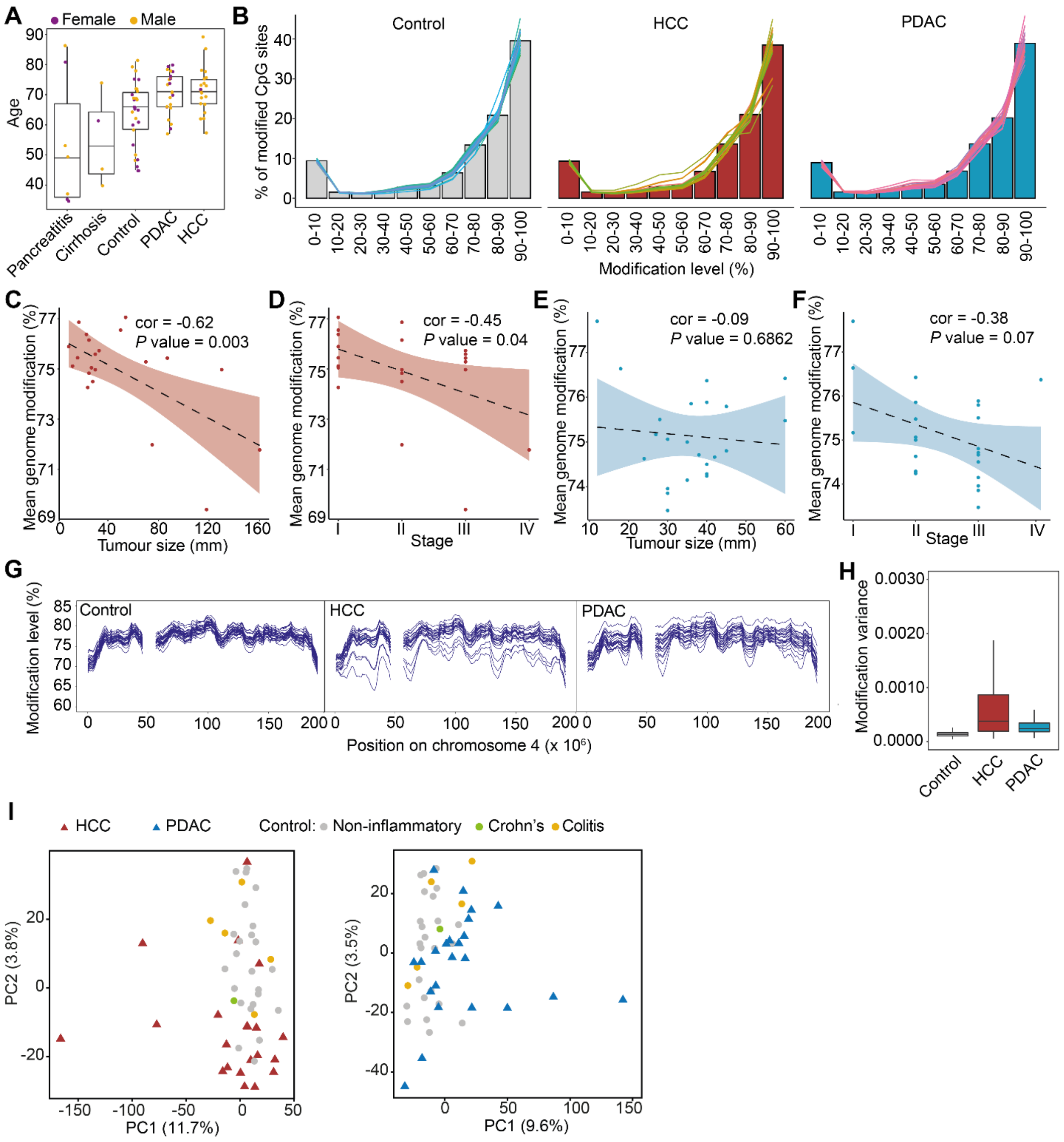

Fig. S2. Global cfDNA methylation patterns in cancer and controls.

(A) Age and gender distribution of pancreatitis, cirrhosis, PDAC, HCC and non-cancer control patients included in cfTAPS cohort. **(B)** Genome-wide distribution of CpG modification in cfDNA in non-cancer controls, HCC and PDAC. Bar plots shows distribution of average CpG modification for each group. Overlaid line plots show CpG methylation distribution in each patient. **(C-D)** Correlation plots of average cfDNA CpG modification level in HCC patients and **(C)** tumor size (mm) and **(D)** tumor stage. **(E-F)** Correlation plots for PDAC patients and **(E)** tumor size (mm) and **(F)** tumor stage. Each dot represents an individual patient. Dashed lines represent the linear trend fitted with linear regression. Shaded area represents 95% confidence intervals of the fitted model. Pearson correlation coefficients (cor) and P values are shown in the plots. **(G)** Distribution

of CpG modification levels over chromosome 4 in cfDNA of non-cancer controls, HCC and PDAC. Each line represents an individual patient. Average CpG modification value was calculated per 1 Mb windows along chromosome 4 and Gaussian-smoothed (smoothing window size 10). **(H)** Methylation variance in 1 Mb genomic windows in non-cancer controls, HCC and PDAC. **(I)** PCA plot of cfDNA methylation in 1 kb genomic windows in non-cancer controls and HCC, non-cancer controls and PDAC (Crohn's disease and colitis are coloured in green and yellow respectively).

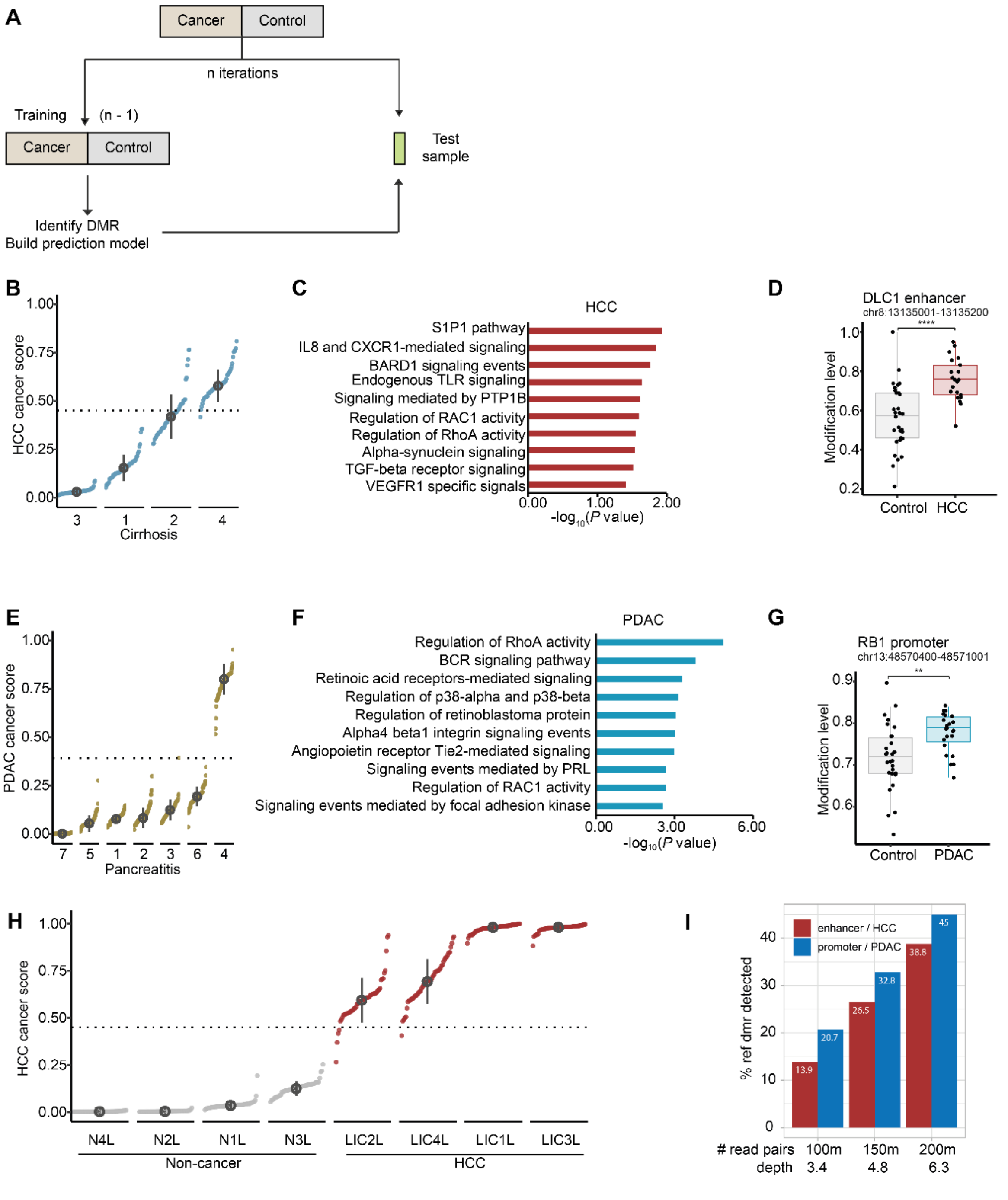

Fig. S3. HCC and PDAC prediction based on cfDNA DMRs.
(A) Overview of the LOO model training and validation approach. Total number of samples is labelled as n. At each iteration, the model training set consists of n – 1 samples. Differentially methylated enhancers (for HCC) or promoters (for PDAC) were selected for model building. The predictive model was evaluated on the held-out test sample in each fold. Cirrhosis and pancreatitis samples were not included in DMR identification and model building. **(B)** HCC cancer prediction scores for cirrhosis samples. Each blue dot represents the predicted score for an individual LOO model. The Black dot shows average probability score for a particular sample. The dashed line

represents probability score threshold. Samples with average probability score above this threshold were predicted as HCC. **(C)** Gene Ontology analysis of genes related to differentially methylated enhancers based in HCC cfDNA (P value < 0.002) using Enrichr *(53)* against NCI-Nature Pathway Interaction. Top 10 categories selected based on P value are shown in the graph. Gene-enhancer interactions were assigned using GeneHancer reference database *(52)*. **(D)** Methylation of representative differentially methylated enhancer in HCC cfDNA for DLC1 gene (two-tailed t-test P value = 8.765e-06). **(E)** PDAC cancer prediction scores for pancreatitis samples. Each yellow dot represents the predicted score for an individual LOO model. The black dot shows the average probability score for a particular sample. The dashed line represents probability score threshold. Samples with average probability score above this threshold were predicted as PDAC. **(F)** Gene Ontology analysis of the genes nearest to the differentially methylated promoters in PDAC cfDNA (P value < 0.002) using Enrichr *(53)* against NCI-Nature Pathway Interaction. Top 10 categories selected based on P value are shown on the graph. **(G)** Methylation of representative differentially methylated promoter in PDAC cfDNA for RB1 gene (two-tailed t-test P value = 0.0017). **(H)** HCC cancer prediction scores for the independent cfDNA WGBS dataset (EGAD00001004317) *(24)*. Each dot represents the predicted score for an individual LOO model. Grey dot belongs to non-cancer controls and the red dot belongs to HCC. The Black dot shows average probability score for a particular sample. The dashed line represents probability score threshold. Samples with average probability score above this threshold were predicted as HCC. **(I)** Percentage of ref DMRs that can be detected in down-sampled reads. DMRs that were identified in original LOO model training were treated as ref DMRs.

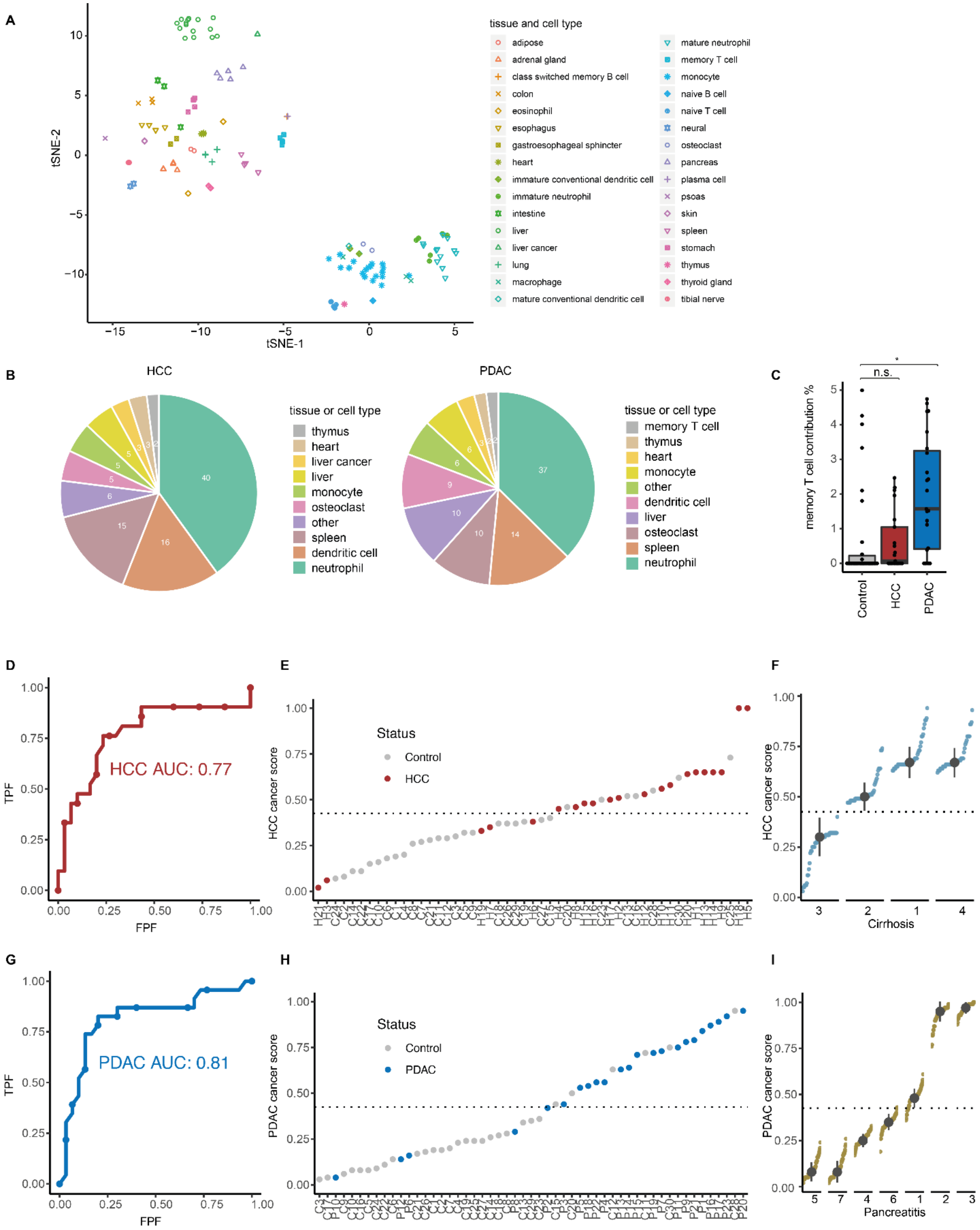

Fig. S4. cfDNA tissue of origin.

(A) t-SNE plot of reference tissue methylation atlas. **(B)** The average tissue contribution in HCC and PDAC individuals. **(C)** Boxplot showing the estimated T cell contribution in non-cancer, HCC

and PDAC cfDNA samples. **(D)** ROC curve of model performance using tissue contribution to classify HCC vs. non-cancer. **(E)** LOO cancer prediction scores for HCC and non-cancer controls using classifiers trained on tissue contribution. The dashed line represents the probability score threshold. Samples with probability score above this threshold were predicted as HCC. **(F)** Cancer scores for cirrhosis samples using HCC vs. non-cancer classifiers. Each blue dot represents the predicted scores for an individual model. Black dot shows the average probability score for a particular sample. Dashed line represents probability score threshold. Samples with average probability score above this threshold were predicted as HCC. **(G)** ROC curve of model performance using tissue contribution to classify PDAC vs control. **(H)** LOO cancer prediction scores for PDAC and non-cancer controls using classifiers built based on tissue contribution. Dashed line represents probability score threshold. Samples with probability score above this threshold were predicted as PDAC. **(I)** PDAC Cancer scores for pancreatitis samples using PDAC vs. non-cancer classifiers. Each yellow dot represents the predicted scores for an individual model. Black dot shows the average probability score for a particular sample. Dashed line represents probability score threshold. Samples with average probability score above this threshold were predicted as PDAC.

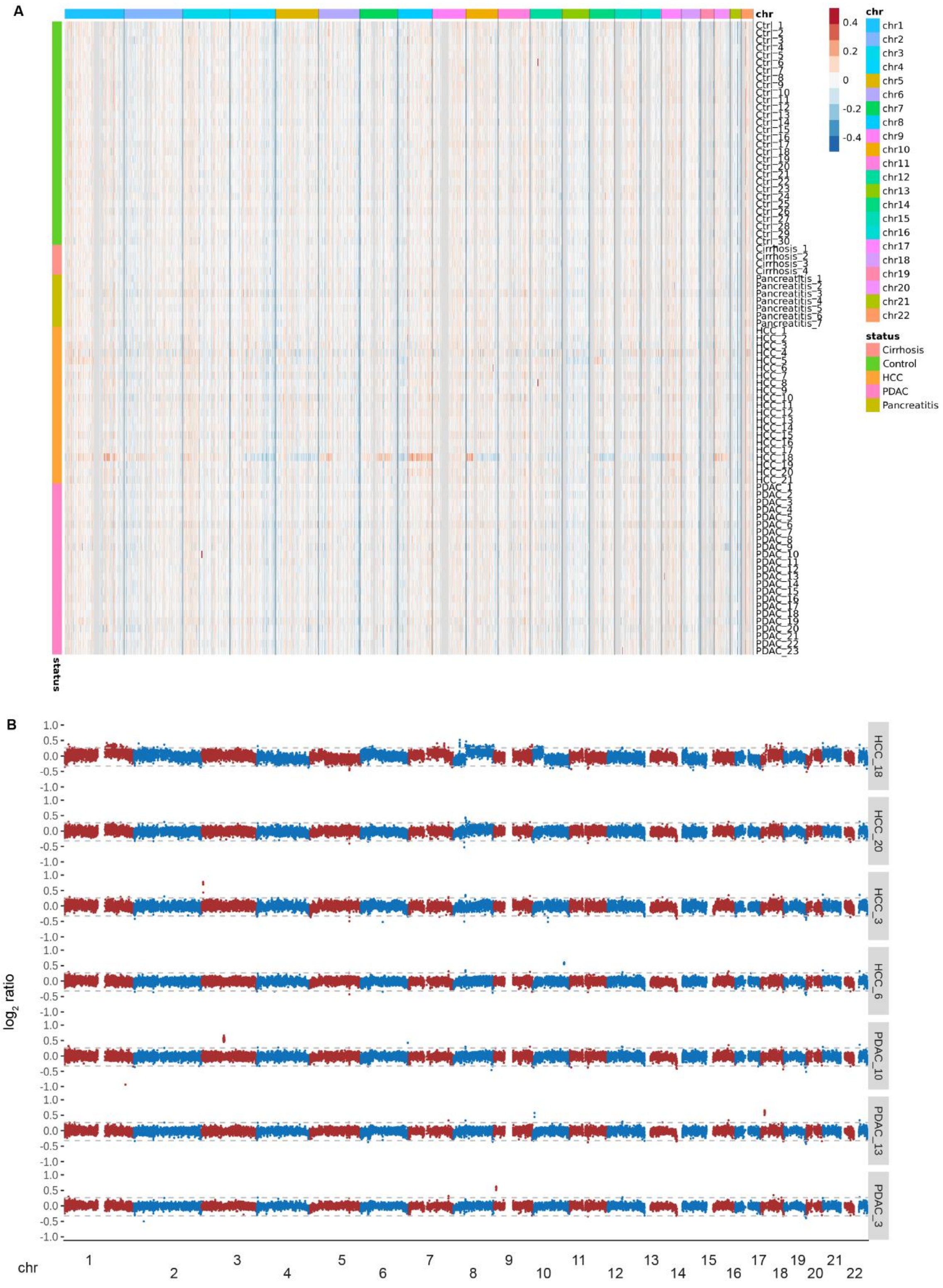

Fig. S5. CNVs analysis in cfDNA.

(A) CNV estimation heatmap from cfDNA in 100kb bin. **(B)** cfDNA samples with CNV larger than 500k.

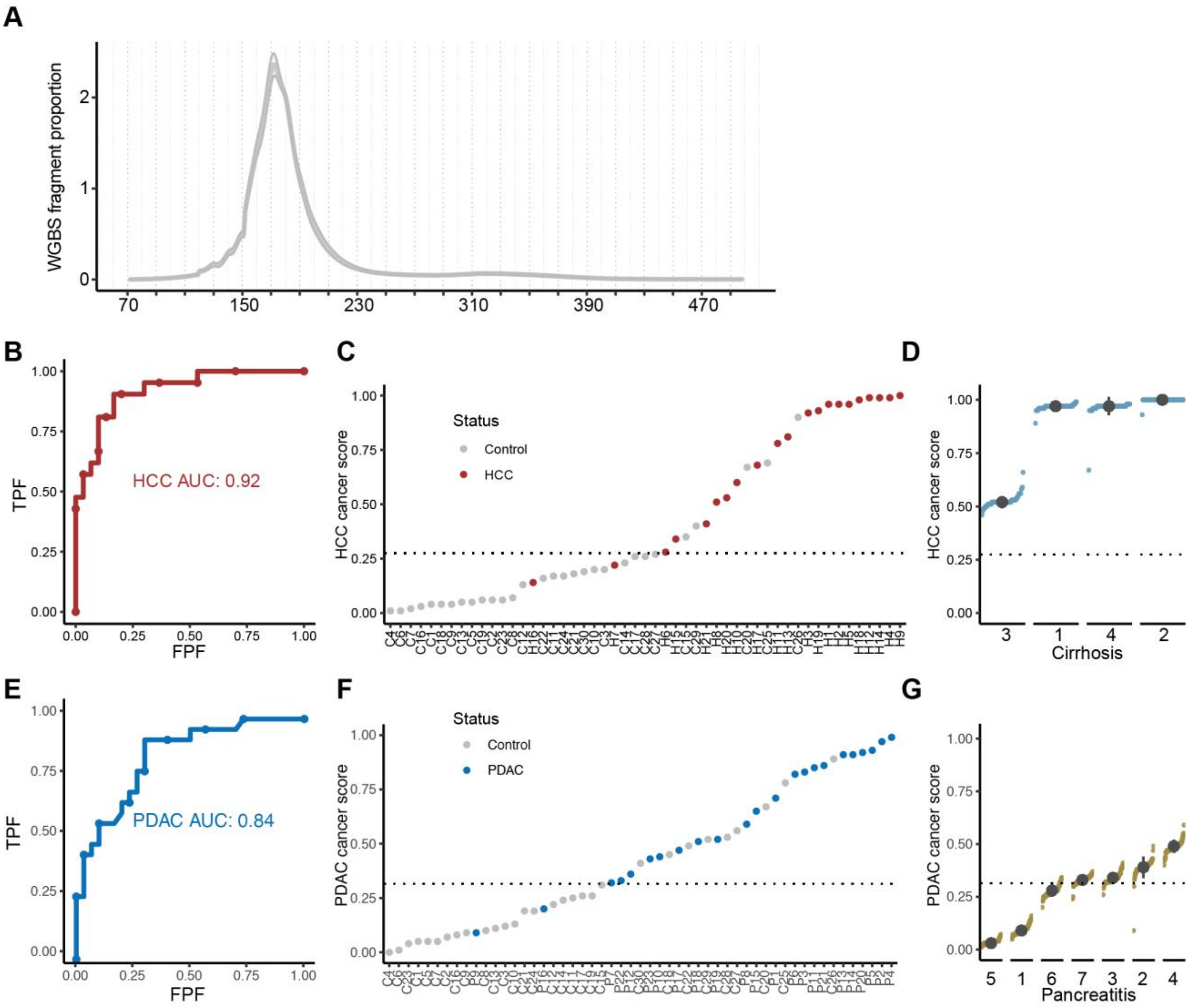

Fig. S6. cfDNA fragmentation patterns for cancer prediction.

(A) Fragment size distribution of cfDNA in public whole genome bisulfite sequencing data *(24)*. Frequency was calculated as number of fragments of particular length divided by total number of fragments. **(B)** ROC curve of HCC and non-cancer control prediction scores from a generalized linear model using proportion of long cfDNA fragments (300-500 bp) in 10 bp bins as features. **(C)** Cancer prediction scores for HCC and non-cancer controls in classifiers trained using LOO cross-validation. The dashed line represents the probability score threshold. Samples with a probability score above this threshold were predicted as HCC. **(D)** HCC cancer prediction scores for cirrhosis samples in these classifiers. Each blue dot represents the predicted score for an individual model. Black dots show average prediction score. The dashed line represents probability score threshold: samples with average probability score above this threshold were predicted as HCC. **(E)** ROC curve of PDAC and non-cancer control prediction scores from a generalized linear model using proportion of long cfDNA fragments (300-500 bp) in 10 bp bins as features. **(F)** LOO cancer prediction scores for PDAC and non-cancer controls in classifiers built based on cfDNA fragments frequency in 10 bp length range. The dashed line represents the probability score threshold. Samples with probability score above this threshold were predicted as PDAC. **(G)**

PDAC cancer prediction scores for pancreatitis samples in classifiers built based on cfDNA fragments frequency in 10 bp length range. Each yellow dot represents the predicted score for an individual model. Black dots show average prediction score. The dashed line represents probability score threshold: samples with average probability score above this threshold were predicted as PDAC.

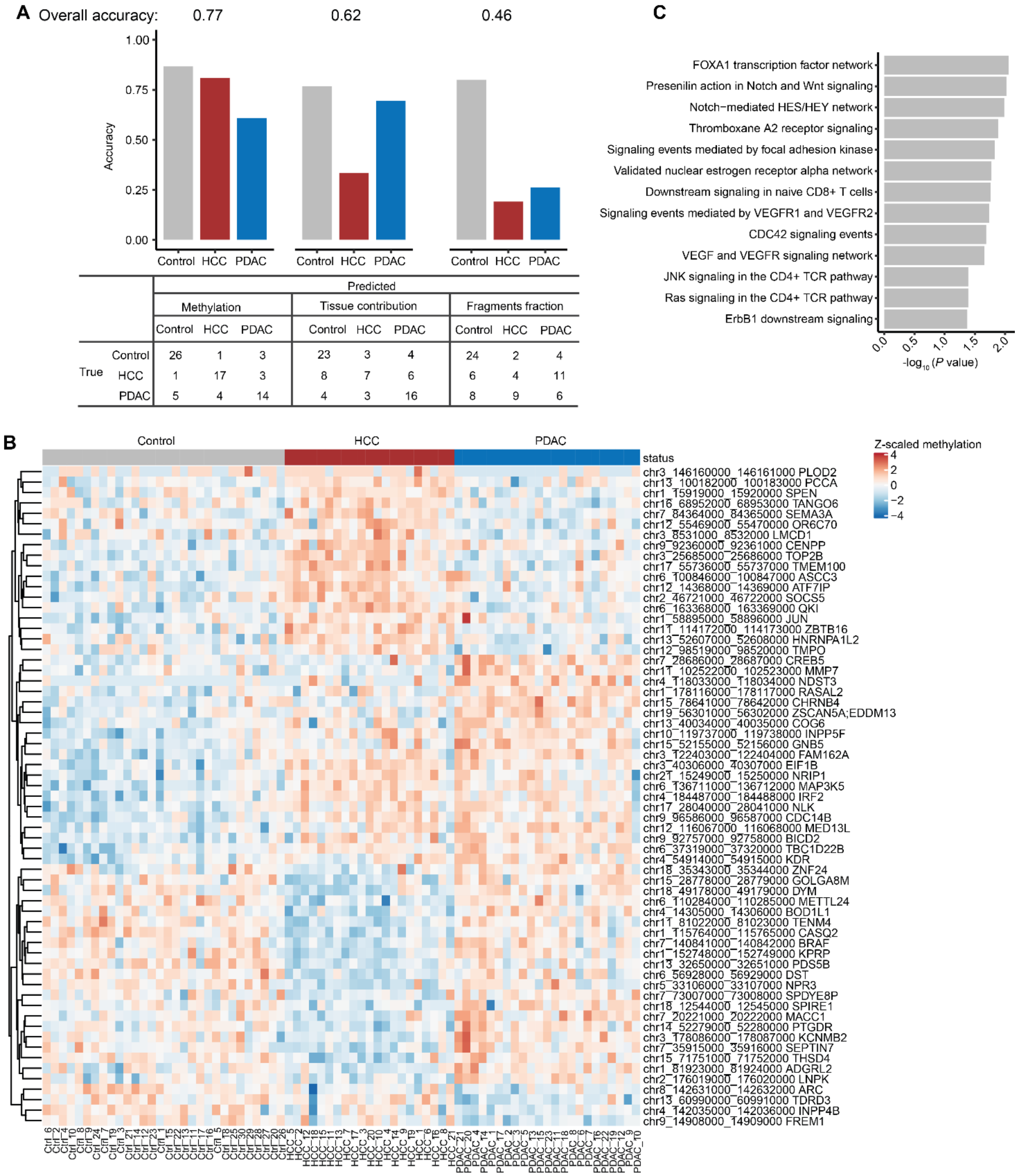

Fig. S7. Multi-cancer detection with cfTAPS.

(A) Methylation, tissue contribution and fragmentation fraction model performance on three-class classification. Upper panel shows the accuracy of each classifier, lower panel shows the actual and predicted patient status in LOO cross-validation analysis. **(B)** Heatmap showing the methylation status of the selected genomic region used for cancer-type prediction. **(C)** Gene Ontology analysis

using Enrichr *(53)* against NCI-Nature Pathway Interaction on the nearest genes of the selected DMRs for three class classification.

Supplementary Tables

Table S1. (Microsoft Excel spreadsheet)

TAPS Sequencing statistics.

Table S2. (Microsoft Excel spreadsheet)

Clinical details of cfTAPS study cohort.

Table S3. (Microsoft Excel spreadsheet)

Differentially methylated enhancers used for HCC vs. Control Prediction.

Table S4. (Microsoft Excel spreadsheet)

Differentially methylated promoters used for PDAC vs. Control Prediction.

Table S5. (Microsoft Excel spreadsheet)

Source of public methylation WGBS data used for generation of tissue map.

Table S6. (Microsoft Excel spreadsheet)

cfDNA tissue contribution for each patient in cfTAPS cohort.

Table S7. (Microsoft Excel spreadsheet)

Fragments length distribution in each individual.

Table S8. (Microsoft Excel spreadsheet)

Methylation Features used for HCC, PDAC and Control Prediction.
